# EARLY COMPREHENSIVE PULMONARY REHABILITATION FOR HOSPITALIZED PATIENTS WITH ACUTE EXACERBATION OF CHRONIC OBSTRUCTIVE PULMONARY DISEASE: A RANDOMIZED CONTROLLED TRIAL

**DOI:** 10.2340/jrm.v56.39953

**Published:** 2024-08-22

**Authors:** Yuqin ZENG, Qian WU, Yan CHEN, Shan CAI

**Affiliations:** 1 Department of Pulmonary and Critical Care Medicine, the Second Xiangya Hospital, Central South University, Changsha, Hunan; 2 Research Unit of Respiratory Disease, Central South University, Changsha, Hunan; 3 Clinical Medical Research Center for Pulmonary and Critical Care Medicine in Hunan Province, Changsha, Hunan; 4 Diagnosis and Treatment Center of Respiratory Disease, Central South University, Changsha, Hunan, China

**Keywords:** pulmonary rehabilitation, acute exacerbation of chronic obstructive pulmonary disease, randomized controlled trial

## Abstract

**Objective:**

To investigate whether an early comprehensive pulmonary rehabilitation intervention initiated during hospital admission is safe and effective for patients with acute exacerbation of chronic obstructive pulmonary disease.

**Design:**

Prospective randomized controlled study.

**Subjects/Patients:**

Patients with acute exacerbation of chronic obstructive pulmonary disease.

**Methods:**

In total, 108 patients were randomized to the early comprehensive pulmonary rehabilitation and usual care groups within 48 hours. The 6-min walking distance, quality of life, breathlessness, and inspiratory muscle strength were measured on admission and discharge. Any adverse events of pulmonary rehabilitation were recorded.

**Results:**

On discharge, the patients in the early comprehensive pulmonary rehabilitation group had a more significant improvement in the 6-min walking distance (47.5 vs 23.0, *p* = 0.04). There was no significant difference in quality of life and breathlessness between the 2 groups. In the early comprehensive pulmonary rehabilitation group, inspiratory muscle strength and peak inspiratory flow were significantly improved, and the changes were much more pronounced than in the usual care group. There were no adverse events.

**Conclusion:**

Early comprehensive pulmonary rehabilitation is safe and effective for hospitalized patients with acute exacerbation of chronic obstructive pulmonary disease, and should be performed during the early stage of hospitalization.

## LAY ABSTRACT

The timing and the mode of pulmonary rehabilitation for acute exacerbation of chronic obstructive pulmonary disease (AECOPD) are controversial. The current study explored the effectiveness and safety of early comprehensive pulmonary rehabilitation within 48 h of hospital admission for patients with AECOPD. A total of 108 hospitalized patients with AECOPD were enrolled and allocated to the early comprehensive pulmonary rehabilitation or usual care group. Their exercise capacity, quality of life, symptoms, and respiratory muscle strength were assessed on admission and discharge. Adverse events such as arrhythmia were recorded. Early comprehensive pulmonary rehabilitation included strength training, inspiratory muscle training, and endurance exercise training. On discharge, the exercise capacity, quality of life, symptoms, and respiratory muscle strength were improved in the pulmonary rehabilitation group. There were no adverse events during pulmonary rehabilitation. However, this rehabilitation did not reduce exacerbations of COPD. In conclusion, it is safe and effective to perform early comprehensive pulmonary rehabilitation for hospitalized patients with AECOPD.

Chronic obstructive pulmonary disease (COPD) is a common, preventable, and treatable disease, characterized by persistent respiratory symptoms and airflow limitations. COPD has a large economic and social burden throughout the world (1). In urban areas of China, the average annual direct medical costs for patients diagnosed with COPD are US$ 30.30 billion. Hospitalization accounts for 56.7% of the total costs (2). Acute exacerbation of COPD (AECOPD) is the main reason for hospitalization; it leads to a decline in lung function, a worsening of symptoms, and impaired physical and psychological well-being (3, 4). Pharmacotherapy and nonpharmacologic therapy are used to relieve an AECOPD and prolong the time to the next event (5).

Pulmonary rehabilitation is a comprehensive intervention to improve the physical and psychological condition of people with chronic respiratory disease (6). Pulmonary rehabilitation plays a key role in the management of COPD and can improve health-related quality of life and exercise capacity (7). A Cochrane review including a total of 20 studies showed that pulmonary rehabilitation improved the quality of life and the exercise capacity of patients after an AECOPD (8). A review suggests that early pulmonary rehabilitation can reduce mortality as well as the number of days in hospital and readmissions (9). On the other hand, a large randomized controlled trial reported that early rehabilitation did not reduce the risk of subsequent readmission, and it actually increased the mortality (10). The 2017 the European Respiratory Society and American Thoracic Society (ERS/ATS) guidelines recommend that pulmonary rehabilitation should be carried out within 3 weeks of hospital discharge and not initiated during hospitalization in patients with AECOPD (11). Experts from Australia and New Zealand recommend that pulmonary rehabilitation should be undergone within 2 weeks of hospital discharge in patients with AECOPD (12). One study suggests that pulmonary rehabilitation carried out within 1 week of discharge can reduce re-exacerbations (13). Despite these recommendations, the optimal duration, type, and mode of pulmonary rehabilitation for patients with AECOPD are still unclear.

Therefore, we conducted a randomized controlled trial to investigate the effect of early comprehensive pulmonary rehabilitation initiated during hospitalization in patients admitted with an AECOPD. We tested the hypothesis that early comprehensive pulmonary rehabilitation during hospitalization improves the exercise capacity, quality of life, symptoms, and inspiratory muscle strength. Furthermore, we assessed whether early comprehensive pulmonary rehabilitation during hospitalization could reduce subsequent hospital admissions over a 1-year period.

## METHODS

### Study design

This study randomized controlled clinical trial was conducted at the Department of Pulmonary and Critical Care Medicine in the Second Xiangya Hospital of Central South University in China and complied with the Declaration of Helsinki. This trial was approved by the ethics committees of the Second Xiangya Hospital of Central South University (Number 2017-062) and registered in the Chinese Clinical Trial Registry (ChiCTR2200064968) on 24 October 2022. All patients provided written informed consent to participate. Patients with AECOPD were randomized by using the random number generator from the PASW 18.0 software (PASW Inc., Chicago, IL, USA) and allocated to an early comprehensive pulmonary rehabilitation group or a usual care group at a ratio of 1:1 by a research assistant. Due to the personnel required for these assessments and inevitable interactions with patients, it was not possible to fully blind the assessors regarding group allocation.

### Study population

Patients with medically confirmed AECOPD and a diagnosis of COPD (post-bronchodilator forced expiratory volume in 1 s to forced vital capacity [FEV_1_/FVC] < 0.7) according to the Global Initiative for Chronic Obstructive Lung Disease (GOLD) were recruited and randomized within 24 h of hospital admission (1). AECOPD was defined as an acute worsening of respiratory symptoms resulting in the need for treatment with antibiotics and/or steroids (1). Eligible patients with AECOPD were ≥40 years, consented to join the exercise programme, and could complete the walking test. Patients were excluded if they had comorbidities that might prevent them from undertaking an exercise programme (e.g., balance deficits, cerebral or lower limb palsies, musculoskeletal impairment, or cardiac conditions that would prevent independent exercise training), were unwilling to sign the informed consent form, or had cognitive impairment.

### Usual care group

The usual care group underwent standard healthcare from physiotherapists and physicians. Patients received education that covered the progress of COPD, risk factors, advice on smoking cessation, referral for dietetic advice and nutritional support if appropriate, the benefits and importance of daily exercise after discharge, breathing strategies, pacing and energy-conservation techniques to manage activities of daily living, and self-management strategies for coping with an exacerbation of COPD. On discharge, all patients received a professional proposal for home-based pulmonary rehabilitation from the physiotherapists.

### Interventions

*Early comprehensive pulmonary rehabilitation group.* Early comprehensive pulmonary rehabilitation commenced within 48 h of hospital admission. The pulmonary rehabilitation team, consisting of pulmonologists, professional physiotherapists, and nurses, delivered this programme. A comprehensive pulmonary rehabilitation programme was individually prescribed as follow-up.

*Strength training.* Strength training was performed in patients with a Lovett scale score of < 5 (14). The upper extremity exercises were performed as described previously (15). Briefly, dumbbells were used to perform unsupported strength training. Starting with the upper extremities extended and adducted in anatomical position and shoulders externally rotated, patients were asked to flex their elbows simultaneously while maintaining them close to the thorax and holding the dumbbells, and to return to the initial position. A sandbag was used for lower limb resistance training. The patients were asked to lie on the bed, the sandbag was wrapped onto their lower leg, and they lifted their leg off the bed as high as they could. Daily training intensity was initially set at 50% of the patient’s 1 repetition maximum for 3 sets with 10 repetitions per set (16). The modified CR10 Borg scale (4–6 out of 10) was used to guide the intensity (17). If the score was < 4, then the weight and number of repetitions per set was increased.

*Inspiratory muscle training.* Inspiratory muscle strength was recorded with a threshold inspiratory muscle trainer (POWER-breathe International Ltd; Southam, Warwickshire, UK), including maximum inspiratory pressure (PImax), peak inspiratory flow, and maximum inspiratory volume. The inspiratory muscle training was performed using this trainer as described previously (18). Patients were asked to start breathing at a resistance of 50% of PImax for one session with 30 breaths each day during hospitalization; this was then increased incrementally. The modified CR10 Borg Scale (4–6 out of 10) was used to support decisions on changes to the training load.

*Endurance exercise training.* The patients with AECOPD exercised on a cycle ergometer (Quark PFTergo, Cosmed, Italy) combined with walking 5 days per week while in hospital. The cycle ergometer training sessions were performed as described previously: (*i*) warm-up, 3 min of cycling at 30% peak workload (Wpeak); (*ii*) exercise, 15–20 min of cycling progressing from 30% Wpeak to 80% Wpeak; and (*iii*) cool-down consisting of 5 min of cycling from 30% to 0% Wpeak (19). Wpeak was individualized for each patient based on their 6-min walking distance (6MWD) as follows: Wpeak (W) = {[(0.122 × 6 MWD) + (72.683 × height [m])]–117.109} (20). The training session with walking was started at 60% and progressed to 80% walking distance for 15–20 min. The walking distance was calculated based on the 6-min walking test (6MWT). The total duration of endurance exercise training started at 10–15 min and progressed to 30–45 min. Lower limb fatigue and dyspnoea were assessed by using the modified CR10 Borg scale after exercise. If the score was < 4, then the exercise intensity was increased by 5% every week up to a maximum of 100% of Wpeak.

*Education.* The education component was the same as for the usual care group. On discharge, patients received a professional proposal for home-based pulmonary rehabilitation from the physiotherapists.

### Outcomes and measurements

*Primary outcome.* The pre-specified primary outcome was exercise capacity based on the 6MWT, which was performed according to published guidelines along a 30-m corridor (21). The test was performed twice and the longest distance was used for the analysis. At the beginning and end of the 6MWT, oxygen saturation recorded by a pulse oximeter (Omron), heart rate, and dyspnoea assessed with the modified CR10 Borg scale were recorded. Patients receiving oxygen therapy were given supplemental oxygen during the 6MWT. The primary pre-specified outcome was performed within 48 h of hospital admission and on hospital discharge.

*Secondary outcomes.* The secondary outcomes were quality of life based on the COPD Assessment Test (CAT) (22); dyspnoea measured with the modified Medical Research Council scale (mMRC); and the assessment of the inspiratory muscle changes, namely PImax, peak inspiratory flow, and maximum inspiratory volume measured using the inspiratory muscle trainer. The secondary functional outcome measures were recorded within 48 h of hospital admission and on hospital discharge. Any adverse events, such as arrhythmia, stethalgia, and other sudden adverse events during the pulmonary rehabilitation programme were recorded. In addition, the number of moderate (treated with short-acting bronchodilators plus antibiotics and/or oral corticosteroids) and severe (patient required hospitalization or emergency room visit) acute exacerbation after 1 year of follow-up were recorded.

*Measurements.* Pulmonary function was measured by using a MasterScreen™ pulmonary function test system spirometer (Vyaire Medical, Mettawa, IL, USA); and the spirometric measurements met the standards of the ATS and ERS (23). According to the airway limitation severity of FEV_1_, the patients were divided into GOLD 1: FEV_1_ > 80% predicted, GOLD 2: 50% ≤ FEV_1_ < 80% predicted, GOLD 3: 30% ≤ FEV_1_ < 50% predicted, and GOLD 4: FEV_1_ < 30% predicted. According to the history of exacerbations and severity of symptoms by the CAT and mMRC questionnaires, patients were classified into Group A, B, C, or D.

### Sample size calculation

We used an intention-to-treat analysis to assess the primary outcome. To detect a minimally clinically important difference between groups in the 6MWD (24), assuming a standard deviation of the within-group differences at the end of the intervention period of 98 m in both groups with 90% statistical power, the risk of a type I error (α) of < 5% and a dropout rate of 20%, a minimum of 45 patients would need to be included in each group.

### Statistical analyses

All data were collected by 1 research assistant and then analysed by another research assistant who did not participate in the data collection. The continuous data are expressed as mean (standard deviation [SD]) if normally distributed or the median (interquartile range) if not normally distributed. The continuous variables were statistically compared using a *t*-test, and the categorical variables were compared using Wilcoxon’s test. The χ^2^ test was used to compare the proportion of acute exacerbations in each group. A *p*-value of < 0.05 was considered as statistically significant. All analyses were performed using PASW version 18.0.

## RESULTS

### Baseline characteristics

**Fig. 1:**
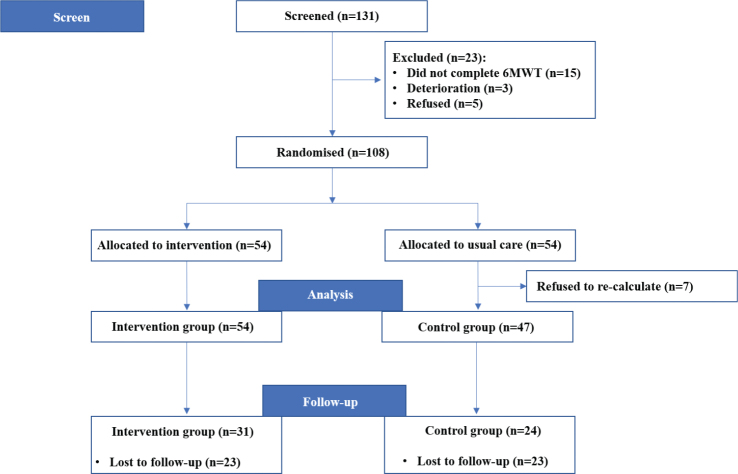
Flow of participants through the study.

Overall, we screened 131 participants. We excluded 23 patients: 15 patients could not complete the 6MWT, 3 patients had a deterioration, and 5 refused to participate. Finally, we recruited 108 participants, with 54 patients in each group, of whom 101 completed this study. We excluded 7 patients in the usual care group from the analysis because they refused to have functional measures reassessed on hospital discharge (Fig. 1). We compared these 7 patients with the other patients in the usual care group and found that they were overweight and had a shorter walking distance (Table SI). There were no significant differences in the other variables.

The demographic and baseline measures are presented in Table I. The 2 groups were comparable in personal characteristics and baseline measures, with no significant differences in 6MWD, Borg scale score, oxygen saturation, heart rate, CAT score, mMRC score, PImax, peak inspiratory flow, and maximum inspiratory volume (*p* > 0.05). At the 1-year follow-up, 46 (45.5%) patients were lost (23 per group). In all, 31 patients in the comprehensive pulmonary rehabilitation group and 24 patients in the usual care group were included in the analysis.

**Table I:** Personal characteristics and baseline measures in each group

Variables	Early rehabilitation (*n* = 54)		Usual care (*n* = 47)	
	Mean/median	SD/IQR	Mean/median	SD/IQR
Age (years)	65.7	8.0	66.8	7.7
Body mass index (kg/m^2^)	21.9	3.6	21.7	3.6
Lung function				
FVC, % predicted	69.6	18.8	70.4	15.8
FEV_1_, % predicted	35.2	13.8	37.3	14.2
FEV_1_/FVC%	39.6	13.0	42.4	14.9
Blood gas assay				
Pondus hydrogenii	7.40	0.04	7.40	0.03
Partial pressure of carbon dioxide	49.52	8.27	48.43	10.62
Partial pressure of oxygen	85.36	24.76	76.47	21.40
Exacerbation history	2	1, 3	2	1, 3
Length of stay (days)	9.3	2.7	9.0	2.8
6-minute walking distance (m)	336.0	117.0	367.4	112.2
Borg scores				
At the beginning of walking test	0.5	0.0, 1.0	0.0	0.0, 1.0
At the end of walking test	4.0	3.0, 4.0	3.0	2.5, 4.0
Oxygen saturation				
At the beginning of walking test	93.3	4.6	94.2	3.7
At the end of walking test	88.4	6.9	90.3	6.6
Heart rate (rpm)				
At the beginning of walking test	95.0	12.8	93.5	13.0
At the end of walking test	107.6	14.8	109.9	14.5
COPD assessment test	19.8	6.7	19.9	7.3
Modified Medical Research Council dyspnoea scale	3	2, 3	2	2, 3
PImax (cmH_2_O)	52.8	19.6	50.6	18.4
Peak inspiratory flow (L/min)	2.96	1.2	2.8	1.1
Maximum inspiratory volume (L)	1.4	0.5	1.2	0.4
Male, *n* (%)	51 (94.4)		44 (93.0)	
GOLD 1–4, n (%)				
Mild	0 (0.0)		1 (2.2)	
Moderate	9 (16.7)		5 (10.9)	
Severe	24 (44.4)		26 (56.5)	
Very severe	21 (38.9)		14 (30.4)	
GOLD B–D, *n* (%)				
B	3 (5.8)		2 (4.9)	
C	3 (5.8)		3 (7.3)	
D	46 (88.4)		39 (87.8)	

GOLD: Global Initiative for Chronic Obstructive Pulmonary Disease; PImax: maximum inspiratory pressure; SD: standard deviation; IQR: interquartile range.

### Primary outcome

**Fig. 2:**
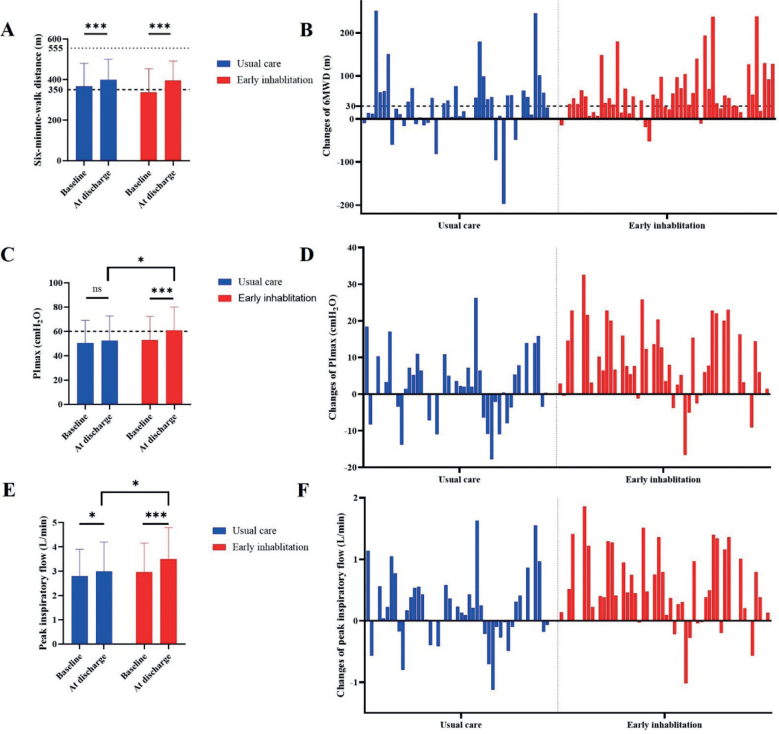
Main outcome measures between the rehabilitation and usual care groups. (A) 6-minute walking distance (6MWD) between the early comprehensive pulmonary rehabilitation group and the usual care group at baseline and on discharge. (B) Changes in the 6MWD between the 2 groups. (C) Maximum inspiratory pressure (PImax) between the 2 groups at baseline and on discharge. (D) Changes in PImax between the 2 groups. (E) Peak inspiratory flow between the 2 groups at baseline and on discharge. (F) Changes in peak inspiratory flow between the 2 groups. ns: no significant difference; *p < 0.05, *** *p* < 0.001.

Most of the patients had a 6MWD below the normal predicted value (555.2 ± 65.3m) at baseline and on discharge. There was no significant difference in the 6MWD at discharge between the early comprehensive pulmonary rehabilitation and the usual care group (*p* > 0.05; Table II). However, in both groups, the 6MWD on discharge was longer than the 6MWD at baseline (394.4 vs 336.0 in the comprehensive pulmonary rehabilitation group, *p* < 0.001 and 399.2 vs 367.4 in the usual care group, *p* < 0.001, respectively). There were more greater changes in the 6MWD in the early comprehensive pulmonary rehabilitation group compared with the usual care group (47.5 vs 23.0, *p* = 0.04) (Table III; Fig. 2A and B). We found that 68.5% (37/54) of patients in the rehabilitation group improved their 6MWD by > 30 m, which is the minimal clinically important difference for patients with COPD. In the usual care group, only 46.8% (22/47) of patients improved their 6MWD by > 30 m. Moreover, 27.7% (12/47) of patients in the usual care group had no improvement or a worse 6MWD on discharge (Fig. 2).

**Table II:** Outcome measures on discharge between rehabilitation and usual care groups

Outcomes	Early rehabilitation (*n* = 54)		Usual care (*n* = 47)		*p*-value
	Mean/median	SD/IQR	Mean/median	SD/IQR	
6-minute walking distance (m)	394.4	96.7	399.2	100.1	0.81
Borg scores					
At the beginning of walking test	0.0	0.0, 1.0	0.0	0.0, 1.0	0.49
At the end of walking test	3.0	2.0, 4.0	3.0	2.0, 3.0	0.37
Oxygen saturation					
At the beginning of walking test	94.7	3.0	94.5	3.2	0.18
At the end of walking test	89.8	6.0	91.1	6.5	0.30
Heart rate (bpm)					
At the beginning of walking test	91.1	13.6	91.3	14.6	0.92
At the end of walking test	103.7	16.6	107.6	16.0	0.24
COPD assessment test	11.9	5.5	13.7	5.2	0.10
Modified Medical Research Council dyspnoea scale	2	1, 2	2	1, 2	0.24
PImax (cmH_2_O)	60.7	19.3	52.6	20.2	0.04*
Peak inspiratory flow (L/min)	3.5	1.3	3.0	1.2	0.03*
Maximum inspiratory volume (L)	1.4	0.5	1.3	0.4	0.41

A *t*-test was used when data were normally distributed; a Mann–Whitney *U* test was used when data were not normally distributed.

* *p* < 0.05.

PImax: maximum inspiratory pressure; SD: standard deviation; IQR: interquartile range.

**Table III:** Changes in outcome measures between rehabilitation and usual care groups

Outcomes	Early rehabilitation (*n* = 54)		Usual care (*n* = 47)		*p*-value
	Median	IQR	Median	IQR	
6MWD (m)	47.5	18.3, 76.3	23.0	–1.0, 61.0	0.04*
Borg scores					
At the beginning of walking test	0.0	0.0, 1.0	0.0	0.0, 1.0	0.98
At the end of walking test	0.5	0.0, 2.0	0.0	0.0, 1.0	0.62
Oxygen saturation					
At the beginning of walking test	1.0	–1.0, 3.0	0.0	–0.1, 2.0	0.20
At the end of walking test	0.5	–2.0, 3.3	0.0	–2.0, 3.0	0.67
Heart rate (bpm)					
At the beginning of walking test	2.0	–4.0, 14.3	3.0	–3.0, 10.0	0.98
At the end of walking test	2.5	–8.5, 17.0	1.0	–5.0, 10.0	0.88
CAT	7.0	4.0, 11.0	5.0	0.0, 11.0	0.09
mMRC	1	0, 2	0	0, 1	0.21
PImax (cmH_2_O)	6.0	0.0, 15.5	0.4	–3.4, 7.2	0.006*
Peak inspiratory flow (L/min)	0.4	0.0, 1.0	0.1	–0.1, 0.4	0.02*
Maximum inspiratory volume (L)	0.0	–0.2, 0.2	0.1	–0.1, 0.4	0.12

A Mann–Whitney *U* test was used.

* *p* < 0.05.

6MWD: 6-minute walking distance; CAT: COPD assessment test; mMRC: modified Medical Research Council dyspnoea scale; PImax: maximum inspiratory pressure; IQR: interquartile range.

### Secondary outcomes

On discharge, PImax and peak inspiratory flow were significantly improved in the early comprehensive pulmonary rehabilitation group compared with the usual care group (PImax 60.7 (19.3) to 52.6 (20.2), *p* = 0.04; peak inspiratory flow 3.5 (1.3) to 3.0 (1.2), *p* = 0.03) (Fig. 2). On discharge, there were no significant differences in Borg scores, oxygen saturation, heart rate, CAT score, mMRC score, and maximum inspiratory volume between the 2 groups (*p* > 0.05; Table II). We found that PImax and peak inspiratory flow increased more in the early comprehensive pulmonary rehabilitation group than in the usual care group: PImax 6.0 (0.0, 15.5) to 0.4 (–3.4, 7.2), *p* = 0.006; peak inspiratory flow 0.4 (0.0, 1.0) vs 0.1 (–0.1, 0.4), *p* = 0.02) (Fig. 2). There were no significant differences in the changes in Borg scores, oxygen saturation, heart rate, CAT score, mMRC score, and maximum inspiratory volume between the 2 groups (*p* > 0.05; Table III). No arrhythmia, stethalgia, or other sudden events occurred during rehabilitation exercises.

There was no significant difference in the number and severity of acute exacerbations after 1 year between the 2 groups (*p* > 0.05; Table IV).

**Table IV:** Numbers of acute exacerbation in both groups after one-year follow up.

Acute exacerbation, *n* (%)	Early rehabilitation (*n* = 31)	Usual care (*n* = 24)	*p*-value
Moderate	2 (6.5)	0 (0)	0.51
Severe	19 (61.3)	13 (54.2)	
Total	21 (67.8)	13 (54.2)	0.30

Both χ^2^ and Fisher’s test were used.

## DISCUSSION

In this randomized controlled trial investigating the effect of early comprehensive pulmonary rehabilitation in patients with AECOPD, exercise capacity, dyspnoea, and quality of life were improved in the early comprehensive pulmonary rehabilitation and usual care groups. The only difference between the 2 groups was a significantly greater improvement in exercise capacity for the early comprehensive pulmonary rehabilitation group. Surprisingly, we also observed a significantly greater improvement in PImax in the early comprehensive pulmonary rehabilitation group compared with the usual care group. There were no adverse events during pulmonary rehabilitation. At the 1-year follow up, the number and severity of acute exacerbations were not significantly different between the 2 groups.

The optimal timing to start a pulmonary rehabilitation programme is controversial. Researchers have started it 24–48 h after hospital admission, after hospital discharge or 2–3 weeks after discharge (25). We started the comprehensive pulmonary rehabilitation programme within 48 h of hospital admission. We found that this timing is safe and effective, consistent with a previous study (26). Most previous pulmonary rehabilitation programmes have combined aerobic exercise and strength training, but we combined strength training, inspiratory muscle training, endurance exercise training, and education. This may be a more suitable programme for patients with AECOPD.

A recent review reported no difference in 6MWD between the early comprehensive pulmonary rehabilitation group initiated during hospital admission and the usual care group (9). This finding is different from our study given that we found an improvement in exercise capacity in both groups. The minimal clinically important difference for the 6MWD has been estimated to be 30 m (24). On discharge, the early comprehensive pulmonary rehabilitation programme showed an increase of 47.5 m, much larger than the usual care group (23.0 m). Our findings are consistent with those of Deepak et al. (27), who reported that the 6MWD increased by 37.9 m in patients who received pulmonary rehabilitation compared with those who did not. In other words, early comprehensive pulmonary rehabilitation seems to be more beneficial for the exercise capacity of patients with AECOPD when it begins during hospital admission.

We used the modified CR10 Borg scale and the mMRC to analyse dyspnoea; it was relieved in both groups. There was a greater tendency for improvement in the early comprehensive pulmonary rehabilitation group compared with the usual care group on discharge, but this difference was not significant. Moreover, the quality of life was similar for both groups based on the CAT scores. In a previous study, the authors reported a significant improvement in quality of life with limited additional gains observed in the intervention group (10). This finding is different from the study by Ko et al. (28), who reported that a comprehensive, individualized pulmonary rehabilitation programme could improve dyspnoea and quality of life (as measured with the St George’s Respiratory Questionnaire) compared with the usual care group at 12 months. The inconsistency in the results might be due to the short duration of the intervention. There was a significant decrease in the mean heart rate after pulmonary rehabilitation exercises in this study, which is consistent with a previous study (29).

In a previous study from our team, we found that inspiratory muscle dysfunction is common in patients with COPD (30). In the present study, we also found that the pressure-generating capacity of the inspiratory pump muscles was reduced (PImax < 60 cmH_2_O) in many patients with AECOPD at baseline. Inspiratory muscle training is an effective method to improve inspiratory muscle strength and endurance in patients with inspiratory muscle weakness (31). Inspiratory muscle training used in isolation or combined with aerobic exercise confers benefits across several outcome areas (32). We used inspiratory muscle training combined with other pulmonary rehabilitation programmes. We found that the combined training was associated with significant improvements in inspiratory muscle strength compared with usual care. Therefore, we suggest that inspiratory muscle training is useful when added to whole-body exercise training in individuals with AECOPD.

Our study has some limitations. First, hospital admissions due to exacerbation of COPD in China usually last 7–10 days. The effectiveness of an early comprehensive pulmonary rehabilitation programme is limited because of time constraints: it is performed only during hospital admission, whereas the guidelines recommend that a pulmonary rehabilitation programme lasts for > 8 weeks (6). Nevertheless, we observed a similar increase in exercise capacity, dyspnoea, quality of life, and inspiratory muscle strength. Therefore, we do not think this factor affected the results. Second, the rate of loss to follow-up was a little high and may have caused selection bias, even though we took several measures such as calling the patient’s family members. Third, we collected only the number and severity of acute exacerbations because, in our preliminary experiment, very few patients returned to the hospital for reassessment.

The strengths of our study are the use of a randomized design; an early comprehensive and standardized rehabilitation programme that was administered for hospitalized patients with AECOPD within 48 hours of admission; inspiratory muscle training as an adjunct to whole-body exercise, which is effective for patients with AECOPD; and a 1-year follow-up.

In conclusion, we found that usual care with normal treatments and education benefited the exercise capacity, dyspnoea, and quality of life for patients with AECOPD. Moreover, early comprehensive pulmonary rehabilitation after AECOPD is safe and seems to accelerate recovery of exercise capacity and inspiratory muscle strength. However, early comprehensive pulmonary rehabilitation did not have an effect on the number and severity of acute exacerbations after 1 year.

## Supplementary Material


