# Supplementary material for: EARLY COMPREHENSIVE PULMONARY REHABILITATION FOR HOSPITALIZED PATIENTS WITH ACUTE EXACERBATION OF CHRONIC OBSTRUCTIVE PULMONARY DISEASE: A RANDOMIZED CONTROLLED TRIAL
Source: J Rehabil Med. 2024 Aug 22;56:39953. doi: 10.2340/jrm.v56.39953 (PMC11358843; doi:10.2340/jrm.v56.39953)
Supplement: Supplementary file 1 [file JRM-56-39953-s1.pdf]

Supplementary material has been published as submitted. It has not been copyedited, or typeset by Journal of Rehabilitation Medicine

Table SI. Personal characteristics and baseline measures in each group

Variables	Complete reassessment (n=47)		No reassessment (n=7)		*P* value
	Mean/median	SD/IQR	Mean/median	SD/IQR	
Age (years)	66.8	7.7	69.0	9.0	0.487
BMI (kg/m^2)	21.7	3.6	42.0	9.0	<0.001*
Lung function					
FVC, % predicted	70.4	15.8	69.4	21.9	0.881
FEV_1, % predicted	37.3	14.2	39.6	19.3	0.706
FEV_1/FVC%	42.4	14.9	43.7	15.1	0.829
Blood gas assay					
Pondus hydrogenii	7.40	0.03	7.40	0.03	0.543
Partial pressure of carbon dioxide	48.43	10.62	53.1	9.4	0.272
Partial pressure of oxygen	76.47	21.40	68.0	18.0	0.325
Exacerbation history	2	1, 3	2	2, 2	0.977
Length of stay (days)	9.0	2.8	7.2	2.4	0.145
6MWD (m)	367.4	112.2	249.3	88.0	0.010*
Borg scores					
At the beginning of walking test	0.0	0.0, 1.0	0.0	0.0, 2.0	0.771
At the end of walking test	3.0	2.5, 4.0	4.0	(3.0, 5.0	0.117
Oxygen saturation					
At the beginning of walking test	94.2	3.7	91.7	3.4	0.105
At the end of walking test	90.3	6.6	85.0	9.3	0.068
Heart rate (bpm)					
At the beginning of walking test	93.5	13.0	94.6	12.7	0.831
At the end of walking test	109.9	14.5	102.0	16.1	0.189
CAT	19.9	7.3	18.3	3.8	0.559
mMRC	2	2, 3	3	2, 4	0.098
PImax (cmH_2O)	50.6	18.4	52.7	21.3	0.779
Peak inspiratory flow (L/min)	2.8	1.1	2.9	1.3	0.830
Maximum inspiratory volume (L)	1.2	0.4	1.4	0.5	0.323
Male, n (%)	44 (93.0)		7 (100)		0.492

Abbreviations: BMI, body mass index; GOLD, Global initiative for chronic obstructive pulmonary disease; 6MWD, 6-minute walking distance; CAT, COPD assessment test; mMRC, modifed medical research council dyspnea scale; PImax, maximum inspiratory pressure; SD, standard deviation; IQR, inter quartile range. *P<0.05.
